# Supplementary material for: Structural disorder and distinctive motifs in the C-terminal region of the MADS-domain transcription factors are conserved across diverse taxa
Source: PLoS One. 2025 Aug 22;20(8):e0330098. doi: 10.1371/journal.pone.0330098 (PMC12373214; doi:10.1371/journal.pone.0330098)
Supplement: S1 Table — (DOCX) [file pone.0330098.s001.docx]

**Table S1. MADS-domain proteins of Arabidopsis, other plants, and non-plant species that were used in this study.**

| **TAIR_ID** | **UniProt_ID** | **NAME** | **Species** | **Type** | **GenBank_ID** | **RAPDB_ID** | **Eukarya** |
| --- | --- | --- | --- | --- | --- | --- | --- |
| AT2G28700 | F4IIT6 | AGL46 | *A. thaliana* | I |  |  | Plantae, Viridiplantae, Angiospermae  Core Eudicot |
| AT3G05860 | F4J9K3 | AGL45 | *A. thaliana* | I |  |  | Core Eudicot |
| AT5G27130 | F4K2U3 | AGL39 | *A. thaliana* | I |  |  | Core Eudicot |
| AT4G36590 | O23222 | AGL40 | *A. thaliana* | I |  |  | Core Eudicot |
| AT2G34440 | O64703 | AGL29 | *A. thaliana* | I |  |  | Core Eudicot |
| AT1G65330 | O80805 | PHE1 | *A. thaliana* | I |  |  | Core Eudicot |
| AT1G65360 | O80807 | AGL23 | *A. thaliana* | I |  |  | Core Eudicot |
| AT4G02235 | O81421 | AGL51 | *A. thaliana* | I |  |  | Core Eudicot |
| AT5G27050 | Q4PSE3 | AGL101 | *A. thaliana* | I |  |  | Core Eudicot |
| AT2G24840 | Q4PSU4 | AGL61 | *A. thaliana* | I |  |  | Core Eudicot |
| AT2G26880 | Q6DR46 | AGL41 | *A. thaliana* | I |  |  | Core Eudicot |
| AT2G15660 | Q6DR69 | AGL95 | *A. thaliana* | I |  |  | Core Eudicot |
| AT1G22590 | Q7X9H1 | AGL87 | *A. thaliana* | I |  |  | Core Eudicot |
| AT5G26580 | Q7X9H2 | AGL34 | *A. thaliana* | I |  |  | Core Eudicot |
| AT5G49420 | Q7X9H5 | AGL84 | *A. thaliana* | I |  |  | Core Eudicot |
| AT5G26950 | Q7X9H9 | AGL93 | *A. thaliana* | I |  |  | Core Eudicot |
| AT5G27070 | Q7X9N2 | AGL53 | *A. thaliana* | I |  |  | Core Eudicot |
| AT5G27960 | Q7XJK5 | AGL90 | *A. thaliana* | I |  |  | Core Eudicot |
| AT5G26650 | Q7XJK6 | AGL36 | *A. thaliana* | I |  |  | Core Eudicot |
| AT1G65300 | Q7XJK8 | PHE2 | *A. thaliana* | I |  |  | Core Eudicot |
| AT1G29962 | Q7XJK9 | AGL64 | *A. thaliana* | I |  |  | Core Eudicot |
| AT5G27580 | Q7XJL1 | AGL89 | *A. thaliana* | I |  |  | Core Eudicot |
| AT1G46408 | Q9C633 | AGL97 | *A. thaliana* | I |  |  | Core Eudicot |
| AT1G31630 | Q9C6V3 | AGL86 | *A. thaliana* | I |  |  | Core Eudicot |
| AT1G31640 | Q9C6V4 | AGL92 | *A. thaliana* | I |  |  | Core Eudicot |
| AT3G66656 | Q9C836 | AGL91 | *A. thaliana* | I |  |  | Core Eudicot |
| AT1G60880 | Q9C963 | AGL56 | *A. thaliana* | I |  |  | Core Eudicot |
| AT1G72350 | Q9C9D4 | AGL60 | *A. thaliana* | I |  |  | Core Eudicot |
| AT5G38620 | Q9FFV9 | AGL73 | *A. thaliana* | I |  |  | Core Eudicot |
| AT5G06500 | Q9FG20 | AGL96 | *A. thaliana* | I |  |  | Core Eudicot |
| AT5G49490 | Q9FGZ5 | AGL83 | *A. thaliana* | I |  |  | Core Eudicot |
| AT5G58890 | Q9FIM0 | AGL82 | *A. thaliana* | I |  |  | Core Eudicot |
| AT5G39810 | Q9FIW6 | AGL98 | *A. thaliana* | I |  |  | Core Eudicot |
| AT5G39750 | Q9FIX0 | AGL81 | *A. thaliana* | I |  |  | Core Eudicot |
| AT5G48670 | Q9FJK3 | AGL80 | *A. thaliana* | I |  |  | Core Eudicot |
| AT5G60440 | Q9FKK2 | AGL62 | *A. thaliana* | I |  |  | Core Eudicot |
| AT5G65330 | Q9FKQ5 | AGL78 | *A. thaliana* | I |  |  | Core Eudicot |
| AT5G38740 | Q9FKR2 | AGL77 | *A. thaliana* | I |  |  | Core Eudicot |
| AT5G40220 | Q9FL10 | AGL43 | *A. thaliana* | I |  |  | Core Eudicot |
| AT5G40120 | Q9FL19 | AGL76 | *A. thaliana* | I |  |  | Core Eudicot |
| AT5G41200 | Q9FLL0 | AGL75 | *A. thaliana* | I |  |  | Core Eudicot |
| AT1G47760 | Q9FZF2 | AGL102 | *A. thaliana* | I |  |  | Core Eudicot |
| AT1G01530 | Q9LMM8 | AGL28 | *A. thaliana* | I |  |  | Core Eudicot |
| AT1G17310 | Q9LN16 | AGL100 | *A. thaliana* | I |  |  | Core Eudicot |
| AT1G48150 | Q9LNG8 | AGL74 | *A. thaliana* | I |  |  | Core Eudicot |
| AT5G04640 | Q9LZ61 | AGL99 | *A. thaliana* | I |  |  | Core Eudicot |
| AT3G04100 | Q9M8W6 | AGL57 | *A. thaliana* | I |  |  | Core Eudicot |
| AT5G27090 | Q9S9U2 | AGL54 | *A. thaliana* | I |  |  | Core Eudicot |
| AT1G28450 | Q9SGP3 | AGL58 | *A. thaliana* | I |  |  | Core Eudicot |
| AT1G28460 | Q9SGP4 | AGL59 | *A. thaliana* | I |  |  | Core Eudicot |
| AT4G11250 | Q9SUT6 | AGL52 | *A. thaliana* | I |  |  | Core Eudicot |
| AT2G40210 | Q9XEF1 | AGL48 | *A. thaliana* | I |  |  | Core Eudicot |
| AT1G59810 | Q9XIE7 | AGL50 | *A. thaliana* | I |  |  | Core Eudicot |
| AT1G60040 | Q9ZUI9 | AGL49 | *A. thaliana* | I |  |  | Core Eudicot |
| AT1G54760 | Q9ZVL8 | AGL85 | *A. thaliana* | I |  |  | Core Eudicot |
| AT1G69120 | P35631 | AP1 | *A. thaliana* | II |  |  | Core Eudicot |
| AT4G37940 | Q9SZJ6 | AGL21 | *A. thaliana* | II |  |  | Core Eudicot |
| AT5G13790 | Q38847 | AGL15 | *A. thaliana* | II |  |  | Core Eudicot |
| AT2G22540 | Q9FVC1 | SVP | *A. thaliana* | II |  |  | Core Eudicot |
| AT3G57230 | A2RVQ5 | AGL16 | *A. thaliana* | II |  |  | Core Eudicot |
| AT3G58780 | P29381 | SHP1 | *A. thaliana* | II |  |  | Core Eudicot |
| AT2G45660 | O64645 | SOC1 | *A. thaliana* | II |  |  | Core Eudicot |
| AT4G18960 | P17839 | AG | *A. thaliana* | II |  |  | Core Eudicot |
| AT5G15800 | P29382 | SEP1 | *A. thaliana* | II |  |  | Core Eudicot |
| AT1G24260 | O22456 | SEP3 | *A. thaliana* | II |  |  | Core Eudicot |
| AT2G03710 | P29383 | SEP4 | *A. thaliana* | II |  |  | Core Eudicot |
| AT2G42830 | P29385 | SHP2 | *A. thaliana* | II |  |  | Core Eudicot |
| AT2G45650 | P29386 | AGL6/RSB1 | *A. thaliana* | II |  |  | Core Eudicot |
| AT5G20240 | P48007 | PI | *A. thaliana* | II |  |  | Core Eudicot |
| AT5G23260 | Q8RYD9 | AGL32/TT16 | *A. thaliana* | II |  |  | Core Eudicot |
| AT1G77950 | F4I8L6 | AGL67 | *A. thaliana* | II |  |  | Core Eudicot |
| AT4G24540 | O82794 | AGL24 | *A. thaliana* | II |  |  | Core Eudicot |
| AT1G77080 | Q9AT76 | MAF1 | *A. thaliana* | II |  |  | Core Eudicot |
| AT3G02310 | P29384 | SEP2 | *A. thaliana* | II |  |  | Core Eudicot |
| AT4G11880 | Q38838 | XAL2 | *A. thaliana* | II |  |  | Core Eudicot |
| AT5G60910 | Q38876 | FUL | *A. thaliana* | II |  |  | Core Eudicot |
| AT3G57390 | Q9M2K8 | AGL18 | *A. thaliana* | II |  |  | Core Eudicot |
| AT4G09960 | Q38836 | STK | *A. thaliana* | II |  |  | Core Eudicot |
| AT3G54340 | P35632 | AP3 | *A. thaliana* | II |  |  | Core Eudicot |
| AT5G62165 | Q9FIS1 | AGL42 | *A. thaliana* | II |  |  | Core Eudicot |
| AT1G22130 | Q9LM46 | AGL104 | *A. thaliana* | II |  |  | Core Eudicot |
| AT5G10140 | Q9S7Q7 | FLC | *A. thaliana* | II |  |  | Core Eudicot |
| AT3G30260 | Q7X9H6 | AGL79 | *A. thaliana* | II |  |  | Core Eudicot |
| AT1G26310 | Q39081 | CAL | *A. thaliana* | II |  |  | Core Eudicot |
| AT2G22630 | Q38840 | AGL17 | *A. thaliana* | II |  |  | Core Eudicot |
| AT5G65080 | Q683D7 | MAF5 | *A. thaliana* | II |  |  | Core Eudicot |
| AT1G31140 | Q9SA07 | GOA | *A. thaliana* | II | * |  | Core Eudicot |
| AT1G71692 | Q38841 | XAL1 | *A. thaliana* | II |  |  | Core Eudicot |
| AT2G14210 | Q9SI38 | ANR1 | *A. thaliana* | II |  |  | Core Eudicot |
| AT1G77980 | Q1PFC2 | AGL66 | *A. thaliana* | II |  |  | Core Eudicot |
| AT3G61120 | Q38837 | AGL13 | *A. thaliana* | II |  |  | Core Eudicot |
| AT5G51870 | Q9LT93 | AGL71/FYL | *A. thaliana* | II |  |  | Core Eudicot |
| AT5G65050 | Q9FPN7 | MAF2 | *A. thaliana* | II |  |  | Core Eudicot |
| AT4G22950 | O82743 | AGL19 | *A. thaliana* | II |  |  | Core Eudicot |
| AT5G65070 | F4KGH9 | MAF4 | *A. thaliana* | II |  |  | Core Eudicot |
| AT5G51860 | Q9FLH5 | AGL72 | *A. thaliana* | II |  |  | Core Eudicot |
| AT5G65060 | Q9LSR7 | MAF3 | *A. thaliana* | II |  |  | Core Eudicot |
|  | Q9XJ60 | OsMADS50 | *Oryza. sativa japonica* | | OsMADS50 | Os03g0122600 | Plantae, Viridiplantae, Angiospermae  Monocot |
|  | P0C5B2 | OsMADS56 | *Oryza. sativa japonica* | | OsMADS56 | Os10g0536100 | Monocot |
|  | Q6YPG9 | OsMADS60 | *Oryza. sativa japonica* | | OsMADS60 | Os02g0104200 | Monocot |
|  | Q8RU31 | OsMADS21 | *Oryza. sativa japonica* | | OsMADS21 | Os01g0886200 | Monocot |
|  | Q10PZ9 | MADS1_ORYSJ | *Oryza. sativa japonica* | | OsMADS1 | Os03g0215400 | Monocot |
|  | Q9XJ66 | OsMADS22 | *Oryza. sativa japonica* | | OsMADS22 | Os02g0761000 | Monocot |
|  | Q2QT50 |  | *Oryza. sativa japonica* | | OsMADS73 | Os12t0407000 | Monocot |
|  |  |  | *Oryza. sativa japonica* | | OsMADS90 | Os07t0133900 | Monocot |
|  |  |  | *Oryza. sativa japonica* | | OsMADS83 | Os04t0313400 | Monocot |
|  | I6LAQ7 | Ambo_PI | *Amborella. trichopoda* | | XP_006847167 |  | Plantae, Viridiplantae, Angiospermae  Basal angiosperm |
|  |  | Ambo_AP3 | *Amborella. trichopoda* | | XP_011628954.1 | | Basal angiosperm |
|  |  | Ambo_AG | *Amborella. trichopoda* | | NP_001292764 |  | Basal angiosperm |
|  |  | Ambo_AGL9 | *Amborella. trichopoda* | | NP_001292758 |  | Basal angiosperm |
|  |  | Ambo_AGL6 | *Amborella. trichopoda* | | NP_001292762 |  | Basal angiosperm |
|  |  | Mangoi_SOC1 | *Manguifera indica* | | XP_044492366 |  | Plantae, Viridiplantae, Angiospermae  Core Eudicot |
|  |  | Soldul_SOC1 | *Solanum dulcamara* | | XP_055817358 |  | Plantae, Viridiplantae, Angiospermae  Core Eudicot |
|  |  | Soldul_MAF5 | *Solanum dulcamara* | | XP_055826431 |  | Core Eudicot |
|  |  | Soldul_AGL104 | *Solanum dulcamara* | | XP_055835538 |  | Core Eudicot |
|  |  | Soldul_AGL8 | *Solanum dulcamara* | | XP_055829872 |  | Core Eudicot |
|  |  | Selm_TIMADS | *Selaginella moellendorffii* | I | EFJ28430 * |  | Plantae, Viridiplantae, Lycophyte  Selaginellales |
|  |  | Selm_AGL104 | *Selaginella moellendorffii* | | XP_024531123 * |  | Selaginellales |
|  |  | Selm_AGL62 | *Selaginella moellendorffii* | | XP_002994589 |  | Selaginellales |
|  |  | Selm_AGL3 | *Selaginella moellendorffii* | | XP_002981044 |  | Selaginellales |
|  |  | Chlod_SOC1 | *Chlorella desiccata* | II | KAH7621717 * |  | Plantae, Viridiplantae  Chlorophyte |
|  |  | Chab_MIKCc | *Chara braunii* | II | GBG82869 * |  | Plantae, Viridiplantae, Charophyta  Charales |
|  |  | Popt_JOINTLESS | *Populus tremuloides* | II | XP_052304750 |  | Plantae, Viridiplantae, Angiospermae  Core Eudicot |
|  |  | Popt_AGL42 | *Populus tremuloides* | II | XP_002318261.2 | | Core Eudicot |
|  |  | Popt_SVP_X1 | *Populus tremuloides* | II | XP_052310622.1 * | | Core Eudicot |
|  |  | Popt_AGL24_X1 | *Populus tremuloides* | II | XP_052308658.1 * | | Core Eudicot |
|  |  | Popt_FLC_X1 | *Populus tremuloides* | II | XP_052307133.1 | | Core Eudicot |
|  |  | Popt_SOC1_X1 | *Populus tremuloides* | II | XP_024440072.1 | | Core Eudicot |
|  |  | Prad_AGL10 | *Pinus radiata* | II | AKC96434.1 * |  | Plantae, Viridiplantae,  Gymnosperm |
|  | P07249 | ARGR1_YEAST | *Saccaromyces cereviseae* | | 1168490 * |  | Fungi, Ascomycota,  Yeast |
|  | P11746 | MCM1_YEAST | *Saccaromyces cereviseae* | | 126821 |  | Yeast |
|  | P38128 | SMP1_YEAST | *Saccaromyces cereviseae* | | 586313 |  | Yeast |
|  | Q12224 | RLM1_YEAST | *Saccaromyces cereviseae* | | 3024551 |  | Yeast |
|  | Q24535 | SRF_DROME | *Drosophila melanogaster* | | 71153173 |  | Animalia, Arthropoda  Insect |
|  | P40791 | MEF2_DROME | *Drosophila melanogaster* | | 353526326 |  | Insect |
|  | P11831 | SRF_HUMAN | *Homo sapiens* | | 134876 |  | Animalia, Chordata  Mammalia |
|  | Q02078 | MEF2A_HUMAN | *Homo sapiens* | | 1170908 |  | Mammal |
|  | Q02080 | MEF2B_HUMAN | *Homo sapiens* | | 1346514 |  | Mammal |
|  | Q06413 | MEF2C_HUMAN | *Homo sapiens* | | 2500875 |  | Mammal |
|  | Q14814 | MEF2D_HUMAN | *Homo sapiens* | | 2500876 |  | Mammal |
