# Supplementary material for: Structural disorder and distinctive motifs in the C-terminal region of the MADS-domain transcription factors are conserved across diverse taxa
Source: PLoS One. 2025 Aug 22;20(8):e0330098. doi: 10.1371/journal.pone.0330098 (PMC12373214; doi:10.1371/journal.pone.0330098)
Supplement: S2 Table — AD = activation domain. AD, Maybe and Not AD definitions are given based on the PADI (plant activation domain identification). PADI score ≥1 and mean disorder >0.5 are defined as “AD”; PADI score ≥1 and mean disorder de ≤ 0.5 are defined as “Maybe”; PADI <1 are defined as “Not AD”. (DOCX) [file pone.0330098.s002.docx]

Table S2. MADS-domain proteins analyzed by Morffy et al., 2024. AD = activation domain. AD, Maybe and Not AD definitions are given based in the PADI (plant activation domain identification). PADI score >=1 and mean disorder >0.5 are defined as “AD”; PADI score >=1 and mean disorder de <=0.5 are defined as "Maybe"; PADI <1 are defined as "Not AD”.

| MADS type | Gene | AD | Maybe | Not AD |
| --- | --- | --- | --- | --- |
| Type I | AGL86 | 2 | 1 | 28 |
| Type I | AGL92 | 1 | 1 | 41 |
| Type I | AGL97 | 7 | 3 | 13 |
| Type I | AGL74 | 7 | 2 | 18 |
| Type I | AGL49 | 2 | 2 | 21 |
| Type I | AGL56 | 4 | 1 | 13 |
| Type I | PHE2 | 3 | 1 | 18 |
| Type I | PHE1 | 6 | 1 | 18 |
| Type I | AGL23 | 2 | 0 | 16 |
| Type I | AGL94 | 5 | 4 | 23 |
| Type I | AGL30 | 1 | 0 | 35 |
| Type I | AGL61 | 1 | 0 | 23 |
| Type I | AGL46 | 6 | 0 | 19 |
| Type I | AGL48 | 5 | 0 | 29 |
| Type I | AGL45 | 2 | 0 | 21 |
| Type I | AGL51 | 6 | 0 | 11 |
| Type I | AGL52 | 2 | 0 | 28 |
| Type I | AGL40 | 1 | 0 | 21 |
| Type I | AGL99 | 12 | 2 | 16 |
| Type I | AGL34 | 5 | 3 | 24 |
| Type I | AGL36 | 2 | 3 | 29 |
| Type I | AGL93 | 5 | 0 | 21 |
| Type I | AGL53 | 1 | 1 | 24 |
| Type I | AGL39 | 7 | 5 | 16 |
| Type I | AGL105 | 1 | 1 | 26 |
| Type I | AGL73 | 11 | 2 | 19 |
| Type I | AGL77 | 3 | 1 | 36 |
| Type I | AGL81 | 5 | 0 | 28 |
| Type I | AGL98 | 5 | 0 | 25 |
| Type I | AGL76 | 1 | 0 | 35 |
| Type I | AGL43 | 2 | 0 | 28 |
| Type I | AGL75 | 4 | 0 | 26 |
| Type I | AGL80 | 2 | 4 | 24 |
| Type I | AGL84 | 7 | 2 | 22 |
| Type I | AGL47 | 3 | 0 | 22 |
| Type I | AGL82 | 8 | 0 | 19 |
| Type I | AGL78 | 2 | 0 | 30 |
| Type I | AGL50 | 0 | 3 | 22 |
| Type I | AGL55 | 0 | 1 | 16 |
| Type I | AGL29 | 0 | 2 | 13 |
| Type I | AGL96 | 0 | 4 | 18 |
| Type I | AGL90 | 0 | 1 | 28 |
| Type I | AGL83 | 0 | 1 | 25 |
| Type I | AGL62 | 0 | 1 | 25 |
| Type I | AGL28 | 0 | 0 | 22 |
| Type I | AGL100 | 0 | 0 | 19 |
| Type I | AGL65 | 0 | 0 | 36 |
| Type I | AGL87 | 0 | 0 | 14 |
| Type I | AGL58 | 0 | 0 | 15 |
| Type I | AGL59 | 0 | 0 | 16 |
| Type I | AGL102 | 0 | 0 | 16 |
| Type I | AGL60 | 0 | 0 | 19 |
| Type I | AGL33 | 0 | 0 | 8 |
| Type I | AGL57 | 0 | 0 | 18 |
| Type I | AGL103 | 0 | 0 | 35 |
| Type I | AGL91 | 0 | 0 | 15 |
| Type I | AGL35 | 0 | 0 | 19 |
| Type I | AGL101 | 0 | 0 | 9 |
| Type I | AGL54 | 0 | 0 | 16 |
| Type I | AGL89 | 0 | 0 | 20 |
| Type II | SEP3 | 2 | 0 | 21 |
| Type II | CAL | 3 | 2 | 15 |
| Type II | AP1 | 3 | 0 | 17 |
| Type II | AGL67 | 2 | 2 | 19 |
| Type II | AGL66 | 2 | 0 | 29 |
| Type II | SEP2 | 2 | 0 | 19 |
| Type II | SHP1 | 1 | 0 | 24 |
| Type II | AGL19 | 1 | 0 | 18 |
| Type II | AGL15 | 1 | 0 | 23 |
| Type II | GOA | 0 | 3 | 16 |
| Type II | XAL1 | 0 | 3 | 16 |
| Type II | SEP4 | 0 | 3 | 20 |
| Type II | ANR1 | 0 | 1 | 20 |
| Type II | SVP | 0 | 5 | 16 |
| Type II | AGL16 | 0 | 1 | 20 |
| Type II | AGL13 | 0 | 2 | 20 |
| Type II | STK | 0 | 2 | 17 |
| Type II | XAL2 | 0 | 2 | 18 |
| Type II | AGL24 | 0 | 3 | 16 |
| Type II | PI | 0 | 1 | 17 |
| Type II | MAF4 | 0 | 2 | 19 |
| Type II | AGL104 | 0 | 0 | 31 |
| Type II | MAF1 | 0 | 0 | 17 |
| Type II | AGL17 | 0 | 0 | 18 |
| Type II | SHP2 | 0 | 0 | 22 |
| Type II | RSB1 | 0 | 0 | 23 |
| Type II | SOC1 | 0 | 0 | 19 |
| Type II | AGL79 | 0 | 0 | 22 |
| Type II | AP3 | 0 | 0 | 21 |
| Type II | AGL18 | 0 | 0 | 23 |
| Type II | AG | 0 | 0 | 22 |
| Type II | AGL21 | 0 | 0 | 20 |
| Type II | FLC | 0 | 0 | 17 |
| Type II | SEP1 | 0 | 0 | 23 |
| Type II | AGL32 | 0 | 0 | 23 |
| Type II | AGL72 | 0 | 0 | 19 |
| Type II | FYL | 0 | 0 | 20 |
| Type II | FUL | 0 | 0 | 22 |
| Type II | AGL42 | 0 | 0 | 17 |
| Type II | MAF2 | 0 | 0 | 17 |
| Type II | MAF3 | 0 | 0 | 17 |
| Type II | MAF5 | 0 | 0 | 9 |
