# Supplementary material for: Structural disorder and distinctive motifs in the C-terminal region of the MADS-domain transcription factors are conserved across diverse taxa
Source: PLoS One. 2025 Aug 22;20(8):e0330098. doi: 10.1371/journal.pone.0330098 (PMC12373214; doi:10.1371/journal.pone.0330098)
Supplement: S3 Table — These packages are available from CRAN (https://CRAN.R-project.org/) or Bioconductor (Huber et al., 2015). (DOCX) [file pone.0330098.s003.docx]

Table S3. R packages used in this research. These packages are available from CRAN ([https://CRAN.R-project.org/](https://cran.r-project.org/)) or Bioconductor (Huber et al., 2015)

| Tool | Version | Citation |
| --- | --- | --- |
| drawProteins | 1.22.2 | Brennan, P. drawProteins: a Bioconductor/R package for reproducible and programmatic generation of protein schematics [version 1; referees: 2 approved] F1000Research 2018, 7:1105 |
| Bioconductor | 3.20 | Huber, W., Carey, V., Gentleman, R., Anders, S., Carlson, M., Carvalho, B. S., Corrada-Bravo H., Davis S., Gatto, L., et al. (2015) Orchestrating high-throughput genomic analysis with Bioconductor. *Nat Methods* 12, 115–121. https://doi.org/10.1038/nmeth.3252 |
| tidyverse | 2.0.0 | Wickham H, Averick M, Bryan J, Chang W, McGowan LD, François R, Grolemund G, Hayes A, Henry L, Hester J, Kuhn M, Pedersen TL, Miller E, Bache SM, Müller K, Ooms J, Robinson D, Seidel DP, Spinu V, Takahashi K, Vaughan D, Wilke C, Woo K, Yutani H (2019). “Welcome to the tidyverse.” Journal of Open Source Software, *4*(43), 1686. doi:10.21105/joss.01686 https://doi.org/10.21105/joss.01686. |
| seqinr | 4.2-36 | Charif, D. and Lobry, J.R. (2007). SeqinR 1.0-2: a contributed package to the R project for statistical computing devoted to biological sequences retrieval and analysis. In Structural approaches to sequence evolution: Molecules, networks, populations, U. Bastolla and M. Porto and H.E. Roman and M. Vendruscolo, eds. Pp 207-232. Springer Verlag, New York. |
| ggpubr | 0.6.0 | Kassambara A (2023). ggpubr: 'ggplot2' Based Publication Ready Plots.  R package version 0.6.0, https://CRAN.R-project.org/package=ggpubr. |
| rstatix | 0.7.2 | Kassambara A (2023). rstatix: Pipe-Friendly Framework for Basic Statistical Tests_. R package version 0.7.2, https://CRAN.R-project.org/package=rstatix. |
| naniar | 1.1.0 | Tierney N, Cook D (2023). Expanding Tidy Data Principles to Facilitate Missing Data Exploration, Visualization and Assessment of Imputations. Journal of Statistical Software, 105(7), 1-31. doi:10.18637/jss.v105.i07 https://doi.org/10.18637/jss.v105.i07. |
| ggforce | 0.4.2 | Pedersen T (2024). ggforce: Accelerating 'ggplot2'. R package version 0.4.2, https://CRAN.R-project.org/package=ggforce. |
| ggfortify | 0.4.17 | Yuan Tang, Masaaki Horikoshi, and Wenxuan Li. "ggfortify: Unified Interface to Visualize Statistical Result of Popular R Packages." The R Journal 8.2 (2016): 478-489. |
| gginnards | 0.2.0 | Aphalo P (2024). gginnards: Explore the Innards of 'ggplot2' Objects. R package version 0.2.0, https://CRAN.R-project.org/package=gginnards. |
| Biostrings | 2.70.3 | Pagès H, Aboyoun P, Gentleman R, DebRoy S (2024). Biostrings: Efficient manipulation of biological strings. R package version 2.70.3, https://bioconductor.org/packages/Biostrings. |
| ggtree | 3.10.1 | Guangchuang Yu. (2022). Data Integration, Manipulation and Visualization of Phylogenetic Trees (1st edition). Chapman and Hall/CRC. doi:10.1201/9781003279242 |
| tidytree | 0.4.6 | Guangchuang Yu. (2022). Data Integration, Manipulation and Visualization of Phylogenetic Trees (1st edition). Chapman and Hall/CRC. doi:10.1201/9781003279242 |
| treeio | 1.26 | Guangchuang Yu. (2022). Data Integration, Manipulation and Visualization of Phylogenetic Trees (1st edition). Chapman and Hall/CRC. doi:10.1201/9781003279242 |
| readr |  | Wickham H, Hester J, Bryan J (2024). readr: Read Rectangular Text Data. R package version 2.1.5, https://CRAN.R-project.org/package=readr. |
| cowplot | 1.1.3 | Wilke C (2024). cowplot: Streamlined Plot Theme and Plot Annotations for 'ggplot2'. R package version 1.1.3, https://CRAN.R-project.org/package=cowplot. |
| ape | 5.8 | Paradis E, Schliep K (2019). ape 5.0: an environment for modern phylogenetics and evolutionary analyses in R. Bioinformatics, 35, 526-528. doi:10.1093/bioinformatics/bty633. https://doi.org/10.1093/bioinformatics/bty633. |
| phytools | 2.4-4- | Revell, L. J. (2024) phytools 2.0: an updated R ecosystem for phylogenetic comparative methods (and other things). PeerJ, 12, e16505. |
