# Supplementary material for: Structural disorder and distinctive motifs in the C-terminal region of the MADS-domain transcription factors are conserved across diverse taxa
Source: PLoS One. 2025 Aug 22;20(8):e0330098. doi: 10.1371/journal.pone.0330098 (PMC12373214; doi:10.1371/journal.pone.0330098)
Supplement: S5 Table — Sites were mapped on domains, according to UniProt and Liu et al., 2021 definition. See main text for a detailed description. (DOCX) [file pone.0330098.s005.docx]

Table S5. Experimentally tested phosphorylation sites obtained from two different databases Athena (<https://athena.proteomics.wzw.tum.de/master_arabidopsisshiny/>), and EPSD (<https://epsd.biocuckoo.cn/Browse.php>). Sites were mapped on domains, according to the UniProt and Li et al., 2021^1^ definition of domains. See main text for expanded methods description.

| Type | Gene | Database | N-terminal | C-terminal |
| --- | --- | --- | --- | --- |
| I | AGL103 | EPSD | 4 |  |
| I | AGL36 | EPSD | 3 | 3 |
| I | AGL43 | EPSD | 13 |  |
| I | AGL45 | EPSD | 1 |  |
| I | AGL46 | EPSD | 1 |  |
| I | AGL53 | EPSD |  | 1 |
| I | AGL57 | EPSD | 3 | 3 |
| I | AGL60 | EPSD | 2 |  |
| I | AGL75 | EPSD |  | 2 |
| I | AGL77 | EPSD | 20 |  |
| I | AGL78 | EPSD | 1 |  |
| I | AGL93 | EPSD |  | 1 |
| I | AGL96 | EPSD |  | 6 |
| I | PHE2 | EPSD |  | 1 |
| II | AGL12 | EPSD | 2 |  |
| II | AGL15 | EPSD | 2 | 2 |
| II | AGL18 | Athena |  | 1 |
| II | AGL19 | EPSD | 1 |  |
| II | AGL21 | Athena/EPSD | 1 |  |
| II | AGL21 | EPSD |  | 2 |
| II | AGL24 | EPSD |  | 4 |
| II | AGL3 | EPSD | 1 |  |
| II | AGL31 | EPSD | 2 |  |
| II | AGL42 | EPSD | 1 |  |
| II | AGL66 | Athena |  | 1 |
| II | AGL8 | EPSD | 1 |  |
| II | MAF3 | EPSD | 1 |  |
| II | MAF4 | Athena | 1 |  |
| II | MAF5 | EPSD |  | 1 |
| II | SEP 1 | EPSD | 1 |  |
| II | SEP 2 | EPSD | 1 |  |
| II | SEP 4 | Athena | 1 |  |
| II | SHP1 | Athena | 1 | 1 |
| II | SHP2 | Athena | 1 | 1 |

^1^Lai X, Vega-Léon R, Hugouvieux V, Blanc-Mathieu R, van der Wal F, Lucas J, et al. The intervening domain is required for DNA-binding and functional identity of plant MADS transcription factors. Nat Commun. 2021 Dec;12.
