# Supplementary material for: Structural disorder and distinctive motifs in the C-terminal region of the MADS-domain transcription factors are conserved across diverse taxa
Source: PLoS One. 2025 Aug 22;20(8):e0330098. doi: 10.1371/journal.pone.0330098 (PMC12373214; doi:10.1371/journal.pone.0330098)
Supplement: S6 Table — (DOCX) [file pone.0330098.s006.docx]

**Table S6. Functional importance of the C-terminal region in MADS-domain proteins across different species, using experimental evidence, is shown in Figure 7.**

| Citation | Protein | Species | Type of experiment | C-terminal modification | Phenotype | | Gain | Loss | Evidence |
| --- | --- | --- | --- | --- | --- | --- | --- | --- | --- |
| van Dijk et al., 2010 | SVP | *A. thaliana* | Y2H | Mutations at positions SS227-228MF |  | | 3 | 3 | 21 interactions, grown in SD/-Leu, -His |
| Cho et al. 1999 | AP1 | *A. thaliana* | Y1H | Removed at positions 88-256; 155-256; 193-256 |  | |  |  | Grown in SD/-Trp, -His  medium |
| Cho et al. 1999 | AP1 | *A. thaliana* | Y1H: minimal activation domains | Removed at positions 223-256 and 228-244 (are acidic residues) |  | |  |  | Grown in SD/-Trp, -His  medium |
| Egea-Cortines et al., 1999 | GLO | *Antirrhinum majus* | Y2H: SQUA (AD)+ DEF(BD) + GLO | Removed by half the C-terminal |  |  | |  | Not grown in SD/-Trp, -His, -Leu, -A or -LacZ |
| Egea-Cortines et al., 2000 | SQUA | *Antirrhinum majus* | Y2H: DEF (BD) + GLO + SQUA(AD) | Removed by half or completely removed the C-terminal |  |  | |  | Not grown in SD/-Trp, -His, -Leu, -A or -LacZ |
| Mizukami et al., 1996 | AG | *A. thaliana* | transgenic plants | Removed at positions 208-285 | *ag*-like |  | |  |  |
| Martin et al., 1994 | MEF2D | *Homo sapiens* | Y1H | Removed at positions  375*-514,  354*-514, 284-514, 252-513,  153-513  *with alternate exon 286-292 |  |  | |  | Transactivation activity is diminished or lost |
| Sridhar et al., 2006 | AP1 | *A.thaliana* | Y2H: SEU + AP1(C-terminal) | Only C-terminal left |  |  | |  | Grown in SD/-Trp |
| Sridhar et al., 2006 | SEP3 | *A.thaliana* | Y2H: SEU + SEP3(C-terminal) | Only C-terminal left |  |  | |  | Grown in SD/-Trp |

Citations

**van Dijk, A. D., Morabito, G., Fiers, M., van Ham, R. C., Angenent, G. C., & Immink, R. G. (2010).** Sequence motifs in MADS transcription factors responsible for specificity and diversification of protein-protein interaction. *PLoS computational biology*, *6*(11), e1001017.

**Cho, S., Jang, S., Chae, S., Chung, K. M., Moon, Y. H., An, G., & Jang, S. K. (1999).** Analysis of the C-terminal region of Arabidopsis thaliana APETALA1 as a transcription activation domain. *Plant molecular biology*, *40*, 419-429.

**Egea‐Cortines, M., Saedler, H., & Sommer, H. (1999).** Ternary complex formation between the MADS‐box proteins SQUAMOSA, DEFICIENS and GLOBOSA is involved in the control of floral architecture in Antirrhinum majus. *The EMBO journal*.

**Mizukami, Y., Huang, H., Tudor, M., Hu, Y., & Ma, H. (1996).** Functional domains of the floral regulator AGAMOUS: characterization of the DNA binding domain and analysis of dominant negative mutations. *The Plant Cell*, *8*(5), 831-845.

**Martin, J. F., Miano, J. M., Hustad, C. M., Copeland, N. G., Jenkins, N. A., & Olson, E. N. (1994).** A Mef2 gene that generates a muscle-specific isoform via alternative mRNA splicing. *Molecular and cellular biology*, *14*(3), 1647-1656.

**Sridhar, V. V., Surendrarao, A., & Liu, Z. (2006).** APETALA1 and SEPALLATA3 interact with SEUSS to mediate transcription repression during flower development.
