# Supplementary figures and images for: Structural disorder and distinctive motifs in the C-terminal region of the MADS-domain transcription factors are conserved across diverse taxa
Source: PLoS One. 2025 Aug 22;20(8):e0330098. doi: 10.1371/journal.pone.0330098 (PMC12373214; doi:10.1371/journal.pone.0330098)

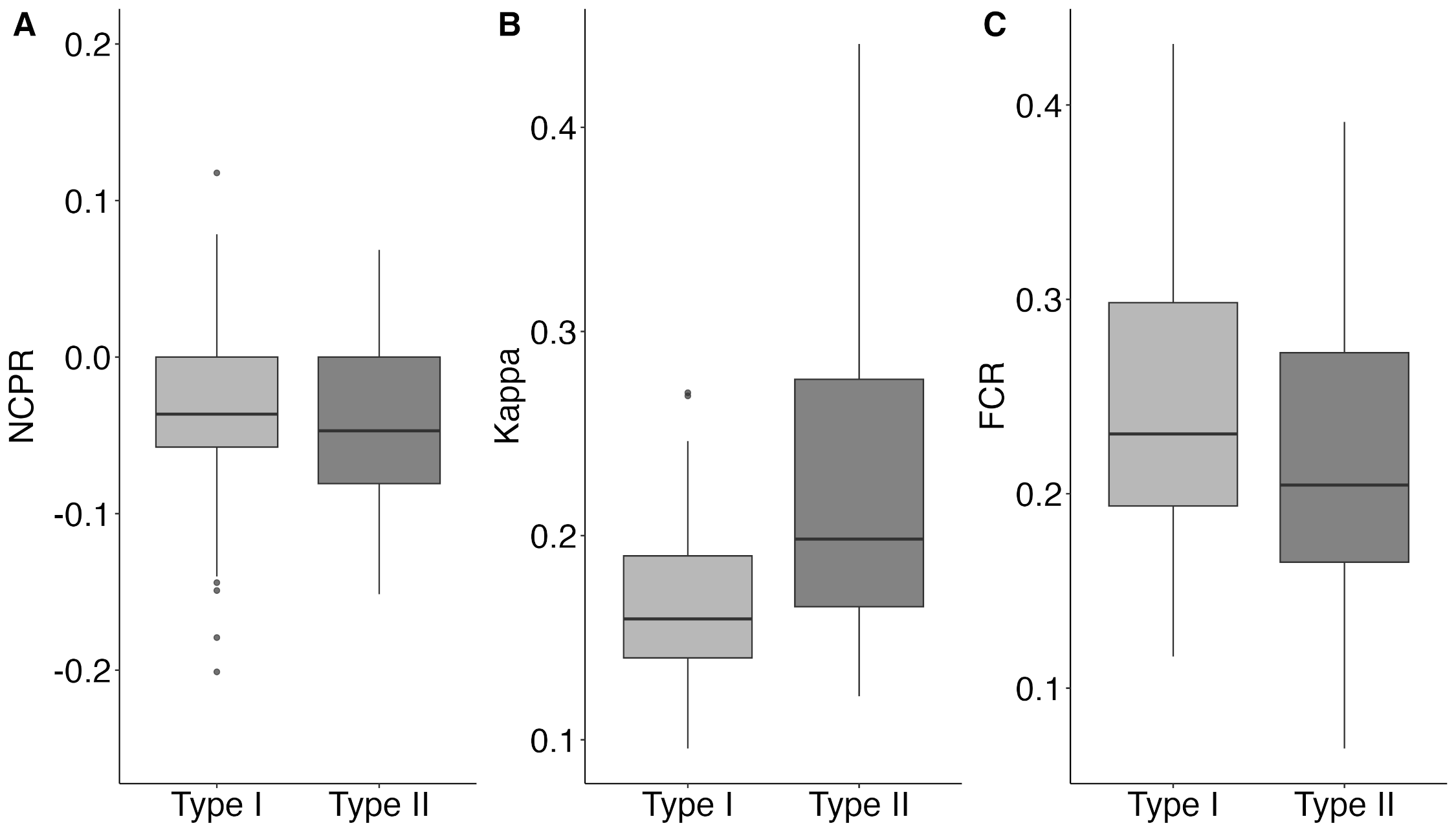

Supplement: S1 Fig — (A) Net charge per residue (NCPR), (B) Charge distribution (Kappa), and (C) Fraction of Charged Residues (FCR). Whiskers indicate ±1.5*IQR based on Tukey test. The middle line represents the median. (TIF) [file pone.0330098.s007.tif]

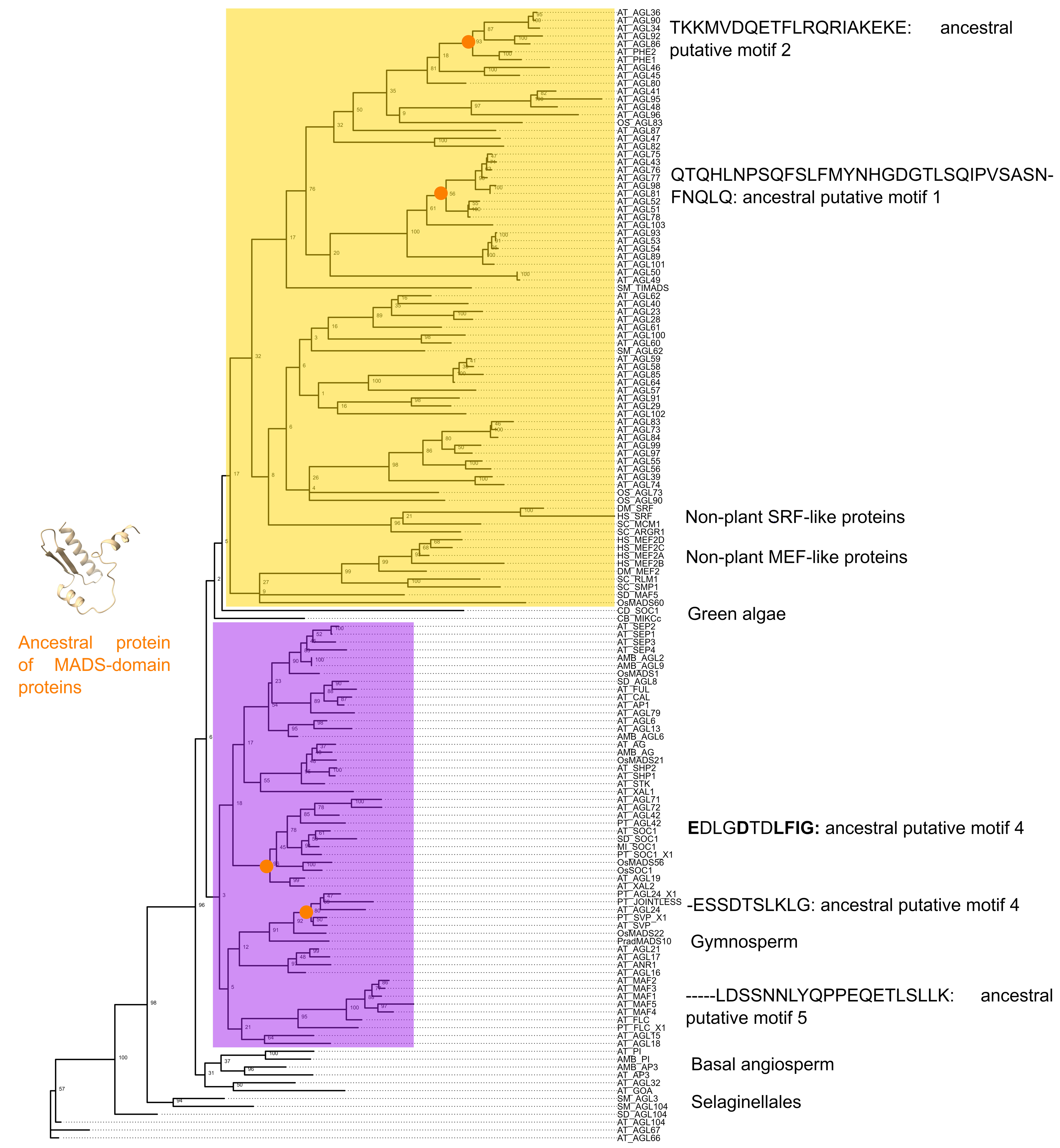

Supplement: S2 Fig — Oryza sativa japonica (Os), Solanum dulcamara (SD), Mangifera indica (MI), Populus trichocarpa (PT), Amborella trichopoda (AMB), Pinus radiata (Prad), Selaginella mollendorffii (SM), Chara braunii (CB), Chlorella dessiccata (CD), Saccharomyces cerevisiae (SC), Drosophila melanogaster (DM), and Homo sapiens (HS). Yellow-shaded branches cover Type I MADS grouped with SRF-like and MEF-like MADS-domain proteins. Purple-shaded branches cover most Type II MADS-domain proteins. Numbers adjacent to nodes represent bootstrap support. Orange dots at particular nodes indicate the putative ancestral motif for that specific clade. (TIF) [file pone.0330098.s008.tif]

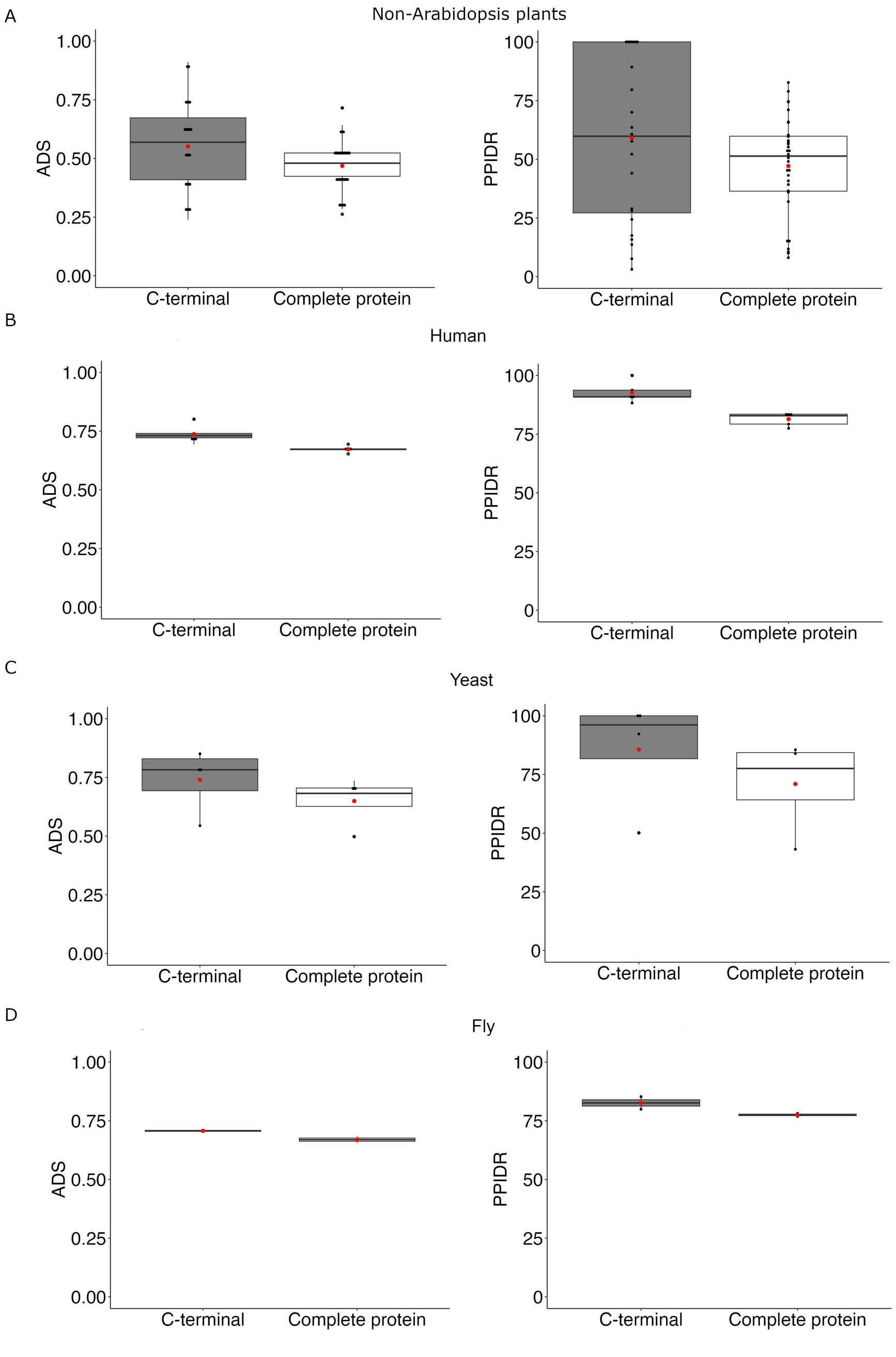

Supplement: S3 Fig — Boxplots showing the ADS and PPIDR values for the C-terminal region and the full-length proteins from: (A) Plant species analysed in this study, excluding Arabidopsis. (B) Saccharomyces cerevisiae, (C) Homo sapiens, and (D) Drosophila melanogaster. Whiskers indicate ±1.5*IQR according to the Tukey test. The middle line represents the median. (TIF) [file pone.0330098.s009.tif]

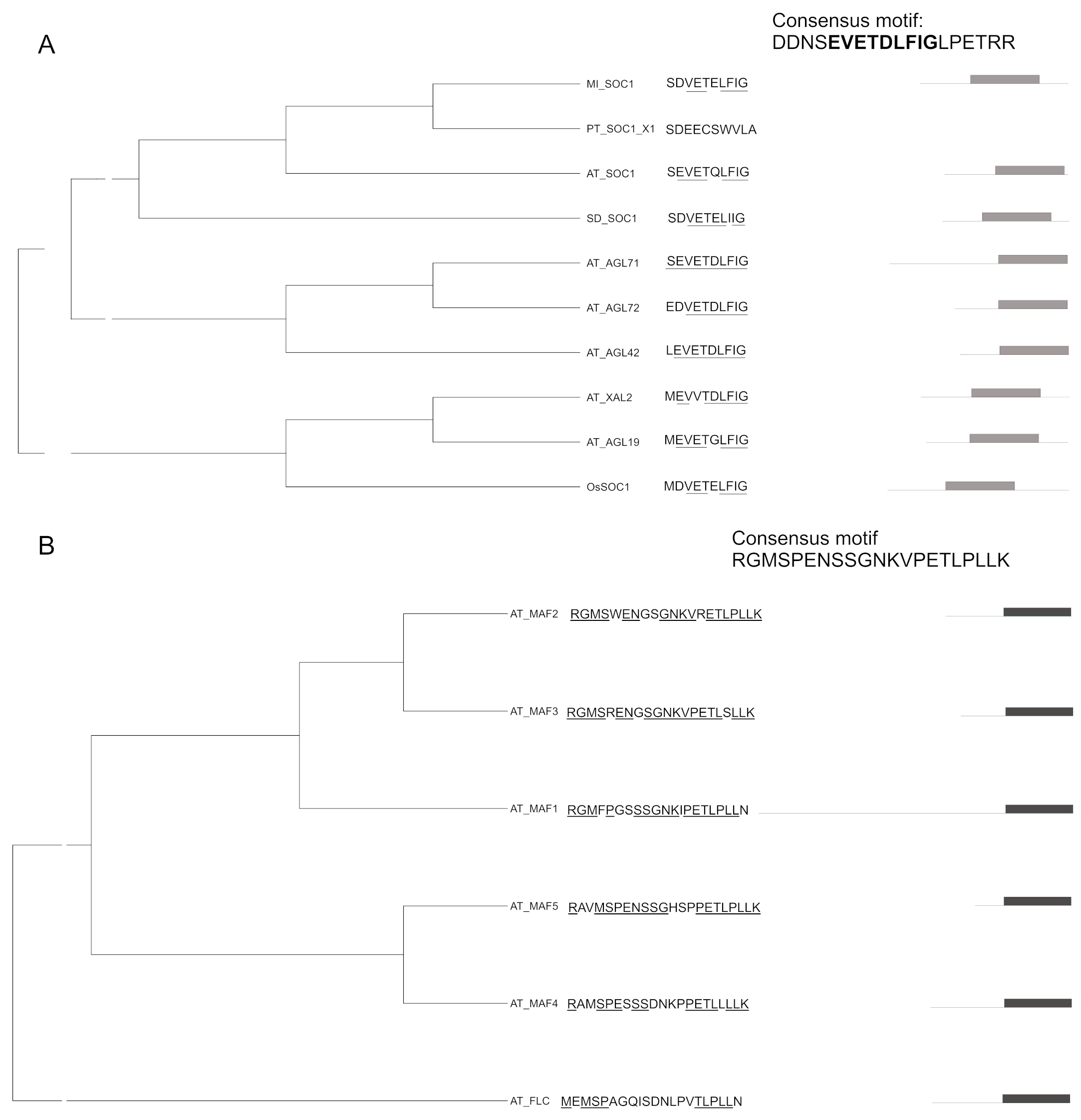

Supplement: S4 Fig — The phylogenetic tree was derived from the complete MADS-domain protein tree shown in Supplementary Fig 2. To enhance the visualization of phylogenetic relationships among the proteins, branch lengths were rescaled and truncated. The conserved amino acid residues of the SOC1-motif and FLC-motif are highlighted in bold within the consensus motif. (TIF) [file pone.0330098.s010.tif]

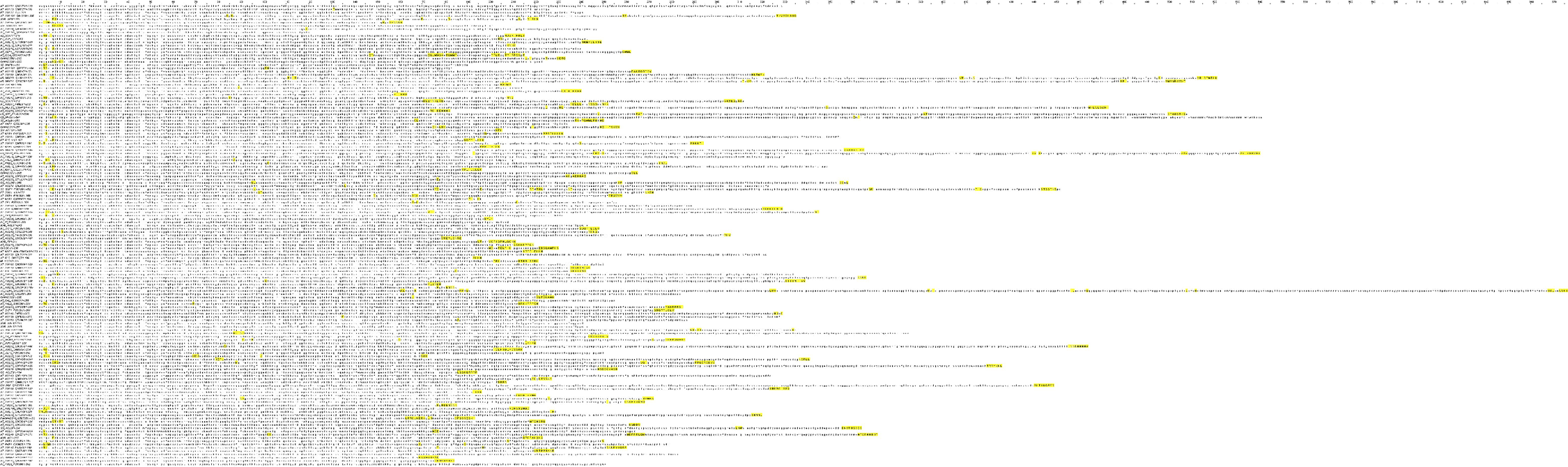

Supplement: S5 Fig — MADS-domain protein sequences from Arabidopsis, other plants, and non-plant organisms are shown, with predicted MoRFs highlighted in yellow Putative MoRFs were identified using the fmoRFpred algorithm [123], based on the analysis of full-length protein sequences. (TIF) [file pone.0330098.s011.tif]
