# Supplementary material for: Structural disorder and distinctive motifs in the C-terminal region of the MADS-domain transcription factors are conserved across diverse taxa
Source: PLoS One. 2025 Aug 22;20(8):e0330098. doi: 10.1371/journal.pone.0330098 (PMC12373214; doi:10.1371/journal.pone.0330098)
Supplement: Attachment — Submitted filename: Comments.pdf [file pone.0330098.s012.pdf]

Structural disorder and distinctive motifs in the C-terminal region of the MADS-domain transcription factors are conserved across diverse taxa

Highlight/ Strengths:

This manuscript presents a bioinformatic analysis of intrinsically disordered regions in MADS-domain transcription factors across diverse taxa. The authors demonstrate that C-terminal disorder is consistently present in both plant and non-plant MADS-domain proteins, with Type II proteins showing higher overall disorder than Type I proteins. They identify conserved motifs within these disordered regions that appear clade-specific, along with overlapping Molecular Recognition Features particularly in Type II proteins. The study further characterizes potential activation domains and liquid-liquid phase separation propensities, finding more activation domains in Type I proteins. While the conservation of these structural features across evolutionary distant taxa strongly suggests functional importance in transcriptional regulation, I have some concerns about the exclusively computational nature of the analysis. The study would benefit from experimental validation of key predictions, clearer distinction between correlation and causality in evolutionary conservation, more robust statistical analysis, and a more integrated model explaining how these various structural features collectively contribute to MADS-domain protein function.

Concern(s)/ Limitations and comments:

- My core concern is about the experimental validation. The study relies exclusively on bioinformatic predictions without any experimental validation of the identified IDRs, MoRFs, or activation domains. While computational approaches provide valuable insights, experimental confirmation is needed to significantly strengthen the findings. I would suggest authors include at least some experimental validation for key findings, particularly for the identified motifs and their potential functional roles. Techniques such as circular dichroism (CD) spectroscopy could confirm the disordered nature of the C-terminal regions, while yeast two-hybrid or co-immunoprecipitation assays could validate protein-protein interactions mediated by the identified MoRFs. Moreover, directed mutagenesis of conserved motifs followed by functional assays would help establish their biological significance, as demonstrated by Cho et al. (1999) for the C-terminal region of APETALA1.
- While the conservation of structural disorder across taxa is interesting, the manuscript doesn't establish whether this conservation reflects functional constraints or simply represents a structural consequence of sequence evolution. Further discussion on this point would strengthen the paper. As noted by Pancsa and Tompa (2012), intrinsic disorder can be maintained through both purifying selection and neutral evolution. The authors could address this by analyzing the rates of synonymous versus non-synonymous substitutions in the disordered regions compared to ordered domains, which would provide evidence for selective

pressure maintaining these IDRs. Additionally, examining the conservation of physicochemical properties rather than just sequence could help distinguish between functional conservation and structural coincidence.

- Although the manuscript speculates on the functional significance of the identified motifs and structural features, it doesn't provide concrete evidence for their roles. I would suggest exploring how these findings connect to known functional differences between Type I and Type II MADS-domain proteins. For instance, the predominance of activation domains in Type I proteins could be related to their specific roles in gametogenesis, while the different patterns of MoRFs in Type II proteins might explain their more diverse developmental functions. Supporting this connection with existing phenotypic data from mutant studies would significantly strengthen the biological relevance of the identified structural features. The recent work by Morffy et al. (2024) on plant transcriptional activation domains could provide a framework for such functional integration.
- While the paper identifies various structural features (IDRs, MoRFs, ADs), it doesn't fully integrate these findings into a cohesive model of how these features might work together to influence protein function. I believe the authors should develop a unified mechanistic model showing how the identified structural elements (IDRs, MoRFs, and ADs) work together to regulate MADS-domain protein function in transcriptional complexes, similar to the framework proposed by Wright and Dyson (2015) for IDR-mediated assembly of transcriptional machinery.
- I believe the comparison between plant and non-plant MADS-domain proteins needs to be expanded to better understand the evolutionary conservation and divergence of these structural features. The current analysis shows that non-plant MADS-domain proteins have longer IDRs, but doesn't explore whether this reflects different functional constraints or adaptations. I suggest including a more detailed phylogenetic analysis that traces the evolution of specific structural features across lineages, similar to the approach taken by Davey et al. (2012) for short linear motifs. This could reveal whether certain structural properties emerged independently in different lineages or were present in the common ancestor of all MADS-domain proteins.
- I believe the statistical analyses need strengthening, especially when comparing Type I and Type II proteins. While Wilcoxon tests are used appropriately, the authors should implement corrections for multiple testing and address how the unequal sample sizes (58 Type I vs. 42 Type II proteins) might affect statistical power. I recommend incorporating bootstrapping or permutation tests to increase confidence in the reported differences, following approaches described by Efron and Tibshirani (1994) for biological data analysis.
