# Supplementary material for: Structural disorder and distinctive motifs in the C-terminal region of the MADS-domain transcription factors are conserved across diverse taxa
Source: PLoS One. 2025 Aug 22;20(8):e0330098. doi: 10.1371/journal.pone.0330098 (PMC12373214; doi:10.1371/journal.pone.0330098)
Supplement: Attachment — Submitted filename: PLOS ONE answers to reviewers.docx [file pone.0330098.s014.docx]

**We thank the reviewers for their valuable comments and feedback, which have undoubtedly helped us improve the manuscript. Thanks to your comments and in the interest of greater clarity, we have decided to include both disorder scores retrieved from the RIDAO platform in the intrinsic disorder analysis: the Average Disorder Score (ADS) and the Percentage of Predicted Disordered Residues (PPDR). In the originally submitted version of the manuscript, we only included the PPDR (MDP), which corresponds to PPDR or Per-MDP in the RIDAO platform. According to RIDAO, PPDR represents the proportion of amino acids with a disorder above 0.5, indicating significant intrinsic disorder, whereas ADS reflects the overall disorder propensity of the protein. As expected, both parameters show a positive correlation. The inclusion of both metrics reinforces the evidence for the intrinsic disorder propensity obtained in the analyzed MADS domain TFs.**

Reviewer #1: The authors have explored the role of intrinsically disordered regions (IDRs) in transcription factors, focusing on MADS-domain proteins in Arabidopsis thaliana. IDRs contribute to protein-protein interactions and functional flexibility. In this study via bioinformatic analysis revealed that most Arabidopsis MADS-domain proteins have IDRs in their C-terminal region, with Type II proteins exhibiting more disorder than Type I. Similar IDRs were found in orthologous proteins from non-plant species (Drosophila, Saccharomyces cerevisiae, and Homo sapiens), often with longer disordered regions. Conserved motifs and putative activation domains were identified in Arabidopsis MADS-domain proteins, suggesting interactions with co-regulatory partners. These findings highlight the evolutionary conservation of structural disorder in MADS-domain proteins and its role in transcriptional regulation.
Comments:
- I think one of the main concerns of the current submission is more editorial. The manuscript was heavily disorganized, especially with figure legends. It was oddly placed and poorly labeled.

**R: Thank you for this observation. The Figure legends were included in the manuscript according to the journal´s instructions. However, as requested by the reviewer, we have included them after the references and have improved their writing to enhance readability. Additionally, we have thoroughly revised the manuscript to improve organization.**

Figure 1) In the box plot showing MDP for Type 1 MAD domain, there is no significant difference between complete protein and C-terminal region like shown in Type 2. The authors have not commented on how that impacts the conclusion on C-terminal IDPs. Does that effect the difference in the protein length and RIDAO Map score.

**R: The lack of differences in the disorder index between the C-terminal domain and the complete Type I MADS-domain proteins reflects the relative proportion of the C-terminal domain within the complete protein. Type I MADS-domain proteins are generally longer than Type II, and their C-terminal regions represent a significantly larger proportion of their total sequence. Therefore, the disorder scores of the C-terminal domain contribute substantially to the overall disorder scores of the entire protein.**

-Figure 2) The pattern of phosphorylation sites in Type 1 and Type 2 different. Phosphorylation can be a key event in stabilizing proteins, and based on the data on Type 2 have higher propensity of phosphorylation in c-terminal and with a small incremental effect. This does not alone support the conclusion that c -terminal of MADS protein have higher phosphorylation site, there should be more analysis done. Comparing non-MADS IDPs can be useful.

**R: We agree with the reviewer’s comment. Analysis of the phosphorylation propensity in a protein sequence does not assure that the predicted sites will be phosphorylated. However, there are several reports supporting that intrinsically disordered regions contain a larger proportion of potential phosphorylation sites than those regions with an ordered and stable structure (Iakoucheva et al. 2004 DOI: 10.1093/nar/gkh253; [Gao and Xu, 2012](https://doi.org/10.1016/j.bpj.2024.10.021)** [**PMID:**](https://doi.org/10.1016/j.bpj.2024.10.021) [**22174266;**](https://pubmed.ncbi.nlm.nih.gov/22174266/) [**Marijn and Ott, 2012**](https://doi.org/10.1016/j.bpj.2024.10.021) [**doi.org/10.3389/fpls.2012.00086; Koike et al., 2019**](https://doi.org/10.3389/fpls.2012.00086) [**doi.org/10.1002/pro.3789;**](https://doi.org/10.1002/pro.3789) **Usher et al., 2024 [https](https://doi.org/10.1016/j.bpj.2024.10.021)**[**://doi.org/10.1016/j.bpj.2024.10.021; Holehouse and Kragelund, 2024**](https://doi.org/10.1016/j.bpj.2024.10.021) [**doi.org/10.1038/s41580-023-00673-0**](http://doi.org/10.1038/s41580-023-00673-0)[**)**](https://doi.org/10.1016/j.bpj.2024.10.021)**. This finding makes sense given the higher exposition of potential phosphorylation sites in more flexible regions. Additionally, it has been found that the amino acid composition, sequence complexity, hydrophobicity, charge, and other sequence features surrounding confirmed phosphorylation sites closely resemble those characteristics of IDRs (Iakoucheva et al. 2004, DOI: 10.1093/nar/gkh253 and by Koike et al., 2019, DOI:10.1002/pro.3789).**

**The analysis suggested by the reviewer with a more extensive set of proteins has been done by Iakoucheva et al. (2004; DOI: 10.1093/nar/gkh253) using different proteomes. These authors have used complete proteomes, finding that proteins containing IDRs show a higher phosphorylation propensity than ordered proteins. We integrated this information into the manuscript (see Discusion, page 19, lines 523 – 542)**

**Because most of the proposed sites have not been experimentally validated, we have not stated that this is the case. Most of the validated phospho-sites are in the MADS-domain proteins' conserved motifs, and these experiments have been conducted under specific developmental stages and/or growth conditions, leaving aside alternative or additional phosphorylations under different experimental conditions. We think that the trend of these analyses is because, in general, the MADS’ IDRs have not received enough attention.**

**This is more evident when we look at the number of validated phosphorylation sites in MADS-domain Type II TFs, the best-characterized of this family. Type II MADS TFs show a similar number of phospho-sites in both the N- and C-terminal regions. This finding contrasts with the data on MADS-domain Type I TFs, where the validated phospho-sites are more abundant in the N-terminal region (see new Supplementary Table S5). Therefore, we think that the data in our manuscript provides an invitation to perform more methodical studies on MADS IDRs to find out the functional relevance of these regions, which are highly represented in this protein family.**

**Additionally, we have added a new paragraph in the phosphorylation results (Page 14, lines 343-351) that compares the experimentally validated phosphorylation patterns between Type I and Type II MADS-domain proteins (see new Supplementary Table S5).**

**We have carefully revised the manuscript, ensuring that the analysis we present in this manuscript refers to propensities, based on validated bioinformatic tools.**

Figure 5) PADI score is dependent on sequence composition, supporting this analysis with different PADI threshold and performance matrix with sensitivity, specificity, ppv, npv with same data can increase the confidence on this analysis.

**R: As mentioned in the manuscript, the data on plant activation domain identification (PADI) were directly obtained from Morffy et al. (2024; DOI: 10.1038/s41586-024-07707-3). In that study, the authors experimentally identified activation domains in various plant TFs using a comprehensive library. This library consisted of overlapping 40 amino acid fragments, spanning the entire set of plant TFs, with a step size of 10 amino acids, resulting in a total of 68,441 fragments. These fragments were screened in yeast to assess their transcriptional activation capacity. Based on this experiment and subsequent normalization, a PADI score was assigned to each fragment. Among the fragments showing transcriptional activation activity, some corresponded to MADS-domain TFs. The PADI scores included in our manuscript are those reported in this study. The localization of activation domains (AD) within MADS-domain TFs was inferred from the results obtained in Morffy et al. (2024), where the authors applied a neural network-based algorithm, known as transcriptional activation domain activity (TADA) network. Their work integrated multiple layers of analysis, including the construction of a feature matrix and the use of methods to assess the impact of both individual input features and border local and global interactions predicted by TADA. Additionally, these results were further analyzed using deep learning to identify key properties relevant to the prediction of ADs. A SHAP analysis was applied to capture non-linear and linear correlations, thereby uncovering complex patterns. This was followed by additional deep-learning steps that culminated in the development of a tool capable of predicting potential ADs. We have included part of this information in the Materials and Methods section. Page 8-9 lines 195-218.**

**As the reviewer may notice, modifying each of the many steps involved in calculating the propensity of specific sequences to function as ADs would be a highly complex task. We consider that such efforts are beyond the scope of the present manuscript.**

- Figure 6) IDPs drive condensate formation and Type 2 have more MDP index and correlation in figure 6 how Type 1 have more LLPS forming propensity than Type 2. The authors have addressed the discrepancy in discussion. It would add more value if there is more similar analysis on known IDPs and LLP forming index.

**R: Three different computational approaches were employed to evaluate the LLPS propensity index: FuzPred, PSPredict, and PSHunter. The table containing the values obtained for the full-length proteins and their corresponding C-terminal regions is included as part of the response to the reviewers (pLLPs software comparison in a reviewer's specific file). Values indicating a high propensity for liquid–liquid phase separation, according to the criteria specified by each prediction tool, are highlighted in yellow.**

**Among these, the analysis performed with FuzPred revealed a higher LLPS propensity in comparison to the other two predictors, although all three identified certain proteins with the potential to form biomolecular condensates.**

**The three tools are designed to predict the likelihood of a protein sequence undergoing liquid-liquid phase separation, particularly in the context of intrinsically disordered regions, but PSPredict and PSHunter exhibit some limitations. Specifically, these methods do not explicitly account for conformational dynamics or contextual interaction features, and they have reduced resolution in capturing the local contributions of specific sequence segments (Chu et al., 2022, doi.org/10.1186/s12859-022-04599-w; Sun et al., 2024, /doi.org/10.1038/s41467-024-46901-9).**

**In contrast, FuzPred incorporates the prediction of conformational fuzziness, referring to the propensity of a disordered region to retain dynamic and structurally adaptable behavior even upon molecular interaction. This approach explicitly considers both multivalency and structural flexibility as key drivers of LLPs, thereby allowing for the identification of functionally relevant interaction-prone regions that may not be detected by more generalized predictors. For this reason, FuzPred was selected as the primary tool for assessing LLPS propensity in our study (Hatos et al., 2023; doi: 10.1093/nar/gkad214).**

Additionally, what if they only input C-terminal region, will that change the LLPS forming Index?

**R: To address the reviewer’s question, which may also be shared by readers, we have included in the revised Figure 6 the data corresponding to the C-terminal regions of the MADS domain TFs. As shown in the updated figure, and as expected, the LLPS propensity indices for these regions are generally higher than those observed for the full-length proteins. This supports the notion that the C-terminal regions contribute significantly to LLPS propensity, and that the rest of the protein sequences may also influence these predictions. Consequently, the LLPS propensity of the full-length proteins does not always correlate with that of their C-terminal regions.**

**As noted in our answer to the previous comment, most LLPS predictors focus on the properties of IDRs, as these regions are key facilitators of protein-protein interactions, some of which drive LLPS through homotypic interactions among disordered segments. Moreover, IDRs can mediate interactions with other proteins, further promoting condensate formation (Xu et al., 2024; doi: 10.1073/pnas.2407633121).**

**However, the capacity to drive LLPS is not determined solely by the disordered nature of the sequence. Instead, it depends on specific sequence features, such as the distribution and patterning of aromatic and charged residues. These sequence-encoded patterns are essential for enabling the multivalent interactions required for the formation of biomolecular condensates (Borcherds et al., 2021; doi: 10.1016/j.sbi.2020.09.004).**

**We have added a comment on this point in the Discussion section of the manuscript (Page 21-22 lines 586-612).**

The discussion is really long; it will help the manuscript if the authors try to keep it more concise in general.

**R: As requested by the reviewer, we have made an effort to reduce the length of the Discussion section. However, this has proven challenging due to the simultaneous request to include additional information.**

**Reviewer #2**: This manuscript presents a bioinformatic analysis of intrinsically disordered regions in MADS-domain transcription factors across diverse taxa. The authors demonstrate that C-terminal disorder is consistently present in both plant and non-plant MADS-domain proteins, with Type II proteins showing higher overall disorder than Type I proteins. They identify conserved motifs within these disordered regions that appear clade-specific, along with overlapping Molecular Recognition Features, particularly in Type II proteins. The study further characterizes potential activation domains and liquid-liquid phase separation propensities, finding more activation domains in Type I proteins. While the conservation of these structural features across evolutionary distant taxa strongly suggests functional importance in transcriptional regulation, I have some concerns about the exclusively computational nature of the analysis. The study would benefit from experimental validation of key predictions, a clearer distinction between correlation and causality in evolutionary conservation, more robust statistical analysis, and a more integrated model explaining how these various structural features collectively contribute to MADS-domain protein function.

Concern(s)/ Limitations and comments:
- My core concern is about the experimental validation. The study relies exclusively on bioinformatic predictions without any experimental validation of the identified IDRs, MoRFs, or activation domains. While computational approaches provide valuable insights, experimental confirmation is needed to significantly strengthen the findings. I would suggest authors include at least some experimental validation for key findings, particularly for the identified motifs and their potential functional roles. Techniques such as circular dichroism (CD) spectroscopy could confirm the disordered nature of the C-terminal regions, while yeast two-hybrid or co-immunoprecipitation assays could validate protein-protein interactions mediated by the identified MoRFs. Moreover, directed mutagenesis of conserved motifs followed by functional assays would help establish their biological significance, as demonstrated by Cho et al. (1999) for the C-terminal region of APETALA1.

**R: We agree with the reviewer’s comment that experimental confirmation would add valuable support to our findings. However, such validation would require a substantial investment of time and resources, which will significantly delay the dissemination of the information presented in this manuscript to the scientific community interested in this important group of TFs. Moreover, this is particularly important because the present study provides, for the first time, evidence that the presence of IDRs in the C-terminal region of MADS-domain TFs is a conserved feature. Furthermore, our results suggest that this structural feature is not exclusive to plant MADS-domain proteins but is conserved across diverse taxa. This finding may therefore be of interest beyond the plant research community, highlighting its broader functional significance and potentially encouraging further investigation by other research groups. Also, it is worth adding that there are published works where the importance of the C-terminal is demonstrated for some Arabidopsis MADS-domain proteins. In particular, specific phenotypes and altered protein-protein interactions in Type II MADS-domain proteins have been associated with point mutations or deletions within IDRs in the C-terminal region, highlighting their functional importance (See new Figure 7 and Table S7; Cho et al., 1999 (DOI: 10.1023/a:1006273127067); Egea-Cortines et al., 1999 (doi: 10.1093/emboj/18.19.5370); Martin et al., 1994 (doi: 10.1128/mcb.14.3.1647); Mizukami et al., 1996 (doi: 10.1105/tpc.9.3.393); van Dijk et al., 2010 (doi:10.1371/journal.pcbi.1001017)). Unfortunately, this has not been the case for Type I MADs-domain proteins, where there is no information regarding the functional characterization of these regions. Whether this lack of information is due to a research bias toward Type II MADS proteins, the difficulty in studying phenotypes in Type I MADS protein mutants, or the apparent specialization of Type I proteins in gametophyte development remains to be determined. We have included part of this information in a new Figure (Fig. 7), a new Supplementary Table 6 and in the Discussion section.**

**Furthermore, we also identified specific motifs and MoRFs within several MADS-domain proteins. For instance, motifs found in SOC1 and FLC are conserved among protein members within their respective clades, and notably, the SOC1 motif is conserved not only within the SOC1 clade but also across different plant species (See Figure S4). We believe this information will encourage and support further experimental studies by researchers working on MADS-domain proteins in diverse biological systems, especially to investigate the functional relevance of these conserved regions. We have included this last paragraph in the Discussion section (Page 23, lines 661-666).**

**Regarding the MADS-domain AP1 protein, Cho et al. (1999; DOI: 10.1023/a:1006273127067) demonstrated that the deletion of the entire C-terminal domain abolished transcriptional activation using the Yeast One Hybrid assay. These authors identified two regions, one proline-rich and the other glutamine-rich, which seem to be important for this function, with the glutamine-rich motif being the most important. This last motif is located near the motif identified in our analysis. This is consistent with previous findings indicating that the functional relevance of a motif depends on its context (Bugge et al., 2020 (doi: 10.3389/fmolb.2020.00110); Holehouse and Kragelund, 2024 (DOI: [10.1038/s41580-023-00673-0](https://doi.org/10.1038/s41580-023-00673-0)).**

- While the conservation of structural disorder across taxa is interesting, the manuscript doesn't establish whether this conservation reflects functional constraints or simply represents a structural consequence of sequence evolution. Further discussion on this point would strengthen the paper. As noted by Pancsa and Tompa (2012), intrinsic disorder can be maintained through both purifying selection and neutral evolution. The authors could address this by analyzing the rates of synonymous versus non-synonymous substitutions in the disordered regions compared to ordered domains, which would provide evidence for selective pressure maintaining these IDRs.

**R: Indeed, a logical next step in the study of intrinsically disordered proteins or regions is to explore their evolutionary patterns. Following the reviewer’s suggestion, we calculated the dN/dS ratio (nonsynonymous to synonymous substitution rate), which reflects the selective pressure acting on substitutions in some Arabidopsis MADS-domain proteins for their N-terminal and the complete proteins (dN/dS_per_CDS in a reviewer's specific folder). Most ratios are higher than 1, indicating positive selection; the N-terminal regions range from 0.84 to 3.01. This suggests that the C-terminal regions, or any other IDRs, may experience weaker negative selection, potentially reflecting a greater tendency toward structural tolerance, as discussed in Pancsa and Tompa (2012; DOI: [10.1371/journal.pone.0034687](https://doi.org/10.1371/journal.pone.0034687)).**

**However, these results must be interpreted with caution. dN/dS estimations are highly sensitive to sequence alignment quality, which is often compromised in highly variable sequence sections, such as the IDRs in the C-terminal region of MADS-domain proteins, due to alignment gaps and homology misinterpretation. Many comparative tools, such as PAML (Yang, 2007; doi.org/10.1093/molbev/msm088), may perform better when these highly divergent regions are excluded to avoid miscalculations (Álvarez-Carretero et al., 2023; doi: 10.1093/molbev/msad041). Moreover, while dN/dS ratios provide insight into selection patterns, IDRs do not evolve independently of the structured domains they are part of, as the functional integrity of the full-length protein must be preserved (Pancsa and Tompa, 2012; DOI: 10.1371/journal.pone.0034687). Ideally, a better approach would be not to estimate dN/dS ratios directly from the corresponding N- or C-terminal regions of the cDNA MSA, but to follow a strategy based on evolutionary distances of IDRs given a particular evolutionary model, as in Zarin et al., 2019 (doi: 10.7554/eLife.46883). Calculating dN/dS from a part of the MSA ignores the physicochemical features of individual residues in a larger context, assuming homology only based on identity.**

Additionally, examining the conservation of physicochemical properties rather than just sequence could help distinguish between functional conservation and structural coincidence.

**R: We agree that evaluating the conservation of net charge per residue (NCPR), charge patterning (kappa), and a fraction of charged residues (FCR) provides valuable insight into how protein evolution influences physicochemical properties. Accordingly, we have added for the reviewer a table with phylogenetic signal calculations on Arabidopsis MADS proteins. However, there are more complex tools to analyze the conservatism of physicochemical properties, where the correlation among traits could be more accurately calculated. Importantly, some evolutionary approaches have attempted to identify conserved molecular features (i. e. NCPR, kappa, FCR, etc.) in the amino acid sequences of IDRs, the findings suggest that, while certain molecular features are linked to known functions in yeast IDRs and may reflect a mechanism of IDR evolution, this pattern does not appear to extend to multicellular organisms like *Drosophila*. These findings indicate that this kind of analysis requires a considerable investment of time and effort. We have included some of this information in the Discussion section.**

- Although the manuscript speculates on the functional significance of the identified motifs and structural features, it doesn't provide concrete evidence for their roles. I would suggest exploring how these findings connect to known functional differences between Type I and Type II MADS-domain proteins. For instance, the predominance of activation domains in Type I proteins could be related to their specific roles in gametogenesis, while the different patterns of MoRFs in Type II proteins might explain their more diverse developmental functions. Supporting this connection with existing phenotypic data from mutant studies would significantly strengthen the biological relevance of the identified structural features. The recent work by Morffy et al. (2024) on plant transcriptional activation domains could provide a framework for such functional integration.

**R: Indeed, it is interesting that Type I MADS-domain proteins exhibit more putative ADs than Type II. In agreement with the reviewer, we also think that this could reflect specific roles for Type I MADS-domain proteins not only in the female gametophyte development but also seed development (Bemer et al., 2010 (DOI: 10.1104/pp.110.160770); Zhang et al., 2023 (DOI: 10.1016/j.celrep.2023.112729). Unfortunately, after reviewing the literature, we were unable to find any study specifically analyzing the functional relevance of the C-terminal domains or any other regions of these proteins. In fact, we only found only a few studies reporting the functional roles of Type I MADS-box genes. For example, a loss of function mutant for *AGL37* (*PHE1*) shows no observable phenotype (Köhler et al., 2003; DOI: 10.1101/gad.257403). We have incorporated this information in Page 22, lines 610-619. Likewise, the *agl23* loss of function mutant exhibits a non-penetrant phenotype in megasporogenesis (Colombo et al., 2008; DOI: 10.1111/j.1365-313X.2008.03485.x). In contrast, single mutants of AGL80 and AGL61 show a maternal-lethal phenotype due to impaired central cell development (Bemer et al., 2010; DOI: 10.1104/pp.110.160770). Interestingly, the nuclear localization of AGL61 depends on its dimerization with AGL80 (Bemer et al., 2008; DOI: 10.1105/tpc.108.058958). AGL62 also interacts with AGL80, and its loss of function mutant results in embryo arrest (Kang et al., 2008; DOI: 10.1105/tpc.107.055137).**

**This outcome adds further interest to the findings presented in this manuscript and encourages future research into the role of the potential ADs in mediating the association between MADS-domain proteins and their co-regulators.**

- While the paper identifies various structural features (IDRs, MoRFs, ADs), it doesn't fully integrate these findings into a cohesive model of how these features might work together to influence protein function. I believe the authors should develop a unified mechanistic model showing how the identified structural elements (IDRs, MoRFs, and ADs) work together to regulate MADS-domain protein function in transcriptional complexes, similar to the framework proposed by Wright and Dyson (2015) for IDR-mediated assembly of transcriptional machinery.

**R: In agreement with the reviewer, we have included in the manuscript a model describing the potential role of specific structural elements within the C-terminal domain of both type I and type II MADS-domain transcription factors, including post-translational modifications (PTMs), molecular recognition features (MoRFs), and activation domains (ADs).**

**The structural flexibility of the C-terminal domain, combined with the presence of MoRFs, ADs, and a high propensity for phosphorylation, suggests a potential regulatory role in modulating the function of MADS-domain transcription factors within transcriptional complexes.**

**In the proposed model, the IDRs of the C-terminal region provide structural flexibility and modularity to MADS transcription factors, facilitating the formation of dynamic complexes. This may occur either through a high propensity for liquid-liquid phase separation (LLPS) or by promoting interactions with other proteins, both of which favor the formation of transcriptional condensates (new Figure 7). In the present study, we describe the presence of MoRFs and ADs within these IDRs located in the C-terminal domain, where the MoRFs may facilitate specific, transient interactions with regulatory proteins or other MADS factors, while in conjunction with the ADs, they can modulate the assembly and stability of transcriptional complexes.**

**It has been reported that the formation of condensates enriched in transcription factors allows for fine-tuned regulation of transcriptional activity, particularly in response to physiological or developmental signals, where diverse proteins can interact with the IDR regions of transcription factors through interactions with their ADs and phase separation (Mann & Notani, 2023 (DOI: 10.1080/19491034.2023.2205758); Boija et al., 2018, doi: 10.1016/j.cell.2018.10.042). We have included a new Figure (Figure 7) with a model and a new Table S6 with the information, explaining the potential role of specific structural elements within the C-terminal domain of both Type I and Type II MADS-domain transcription factors (new Figure 7). Also, we have added some of this information in the Discussion section (Page 23, lines 637-666).**

- I believe the comparison between plant and non-plant MADS-domain proteins needs to be expanded to better understand the evolutionary conservation and divergence of these structural features. The current analysis shows that non-plant MADS-domain proteins have longer IDRs, but doesn't explore whether this reflects different functional constraints or adaptations. I suggest including a more detailed phylogenetic analysis that traces the evolution of specific structural features across lineages, similar to the approach taken by Davey et al. (2012) for short linear motifs. This could reveal whether certain structural properties emerged independently in different lineages or were present in the common ancestor of all MADS-domain proteins.

**R: Thank you for your comment. We agree that a detailed phylogenetic analysis tracing the evolution of specific structural features would significantly improve our understanding of the functional diversification of the MADS-domain protein family. While we recognized the value of the pipeline proposed by Davey et al. (2012; DOI:** [**10.1093/nar/gks854**](https://doi.org/10.1093/nar/gks854)**), the required data preparation and computational resources exceeded the available time for doing it. Instead, we applied more recent bioinformatic approaches to address this question. We performed ancestral sequence reconstruction across the complete phylogeny and mapped amino acid changes, focusing on clades containing the C-terminal motifs identified by MEME. Our analysis revealed that reconstructed ancestral sequences share conserved residues that remain in the derived proteins. These conserved residues may be important for functionally coupling the C-terminal region with other domains of the proteins. However, experimental confirmation will be necessary to validate this hypothesis. We have modified Figure S2 to include the reconstructed ancestral sequences at nodes where MEME motifs were identified.**

- I believe the statistical analyses need strengthening, especially when comparing Type I and Type II proteins. While Wilcoxon tests are used appropriately, the authors should implement corrections for multiple testing and address how the unequal sample sizes (58 Type I vs. 42 Type II proteins) might affect statistical power. I recommend incorporating bootstrapping or permutation tests to increase confidence in the reported differences, following approaches described by Efron and Tibshirani (1994) for biological data analysis.

**R: We agree that employing a permutation-based Wilcoxon test would improve the robustness and reliability of our analysis. To address this, we re-ran the statistical tests using the coin package in R, which implements permutation procedures following the methods described by Strasser and Weber (1999). A detailed explanation of the statistical framework is available in the CRAN documentation and the vignettes associated with it. Overall, most of our results obtained with the permutation-based approach were consistent with our initial analysis, supporting the robustness of our conclusions. The only change we found was for the number of IDRs between Type I and Type II, in lines 300 - 306, p. 12. We have added this new statistical approach in the Materials and Methods (Data analyses and image rendering) section on Pages 10, lines 256-266, and changed statistics tests and p-values in the text.**
